# Turning When Using Smartphone in Persons With and Those Without Neurologic Conditions: Observational Study

**DOI:** 10.2196/41082

**Published:** 2023-03-30

**Authors:** Edoardo Bianchini, Elke Warmerdam, Robbin Romijnders, Klarissa Hanja Stürner, Ralf Baron, Sebastian Heinzel, Francesco Ernesto Pontieri, Clint Hansen, Walter Maetzler

**Affiliations:** 1 Department of Neurology Kiel University Kiel Germany; 2 Department of Neuroscience Mental Health and Sensory Organs (NESMOS) Sapienza University of Rome Rome Italy; 3 Division of Surgery Saarland University Homburg Germany; 4 Faculty of Engineering Kiel University Kiel Germany; 5 Institute of Medical Informatics and Statistics University Hospital Schleswig-Holstein Kiel University Kiel Germany; 6 Santa Lucia Foundation Rome Italy

**Keywords:** turning, turning coordination, smartphone, dual task, dual task cost, Parkinson disease, Parkinson, stroke, multiple sclerosis, low back pain, neurology, neurological, movement, biomechanics, gait, balance, walk, kinesiology, fall

## Abstract

**Background:**

Turning during walking is a relevant and common everyday movement and it depends on a correct top-down intersegmental coordination. This could be reduced in several conditions (en bloc turning), and an altered turning kinematics has been linked to increased risk of falls. Smartphone use has been associated with poorer balance and gait; however, its effect on turning-while-walking has not been investigated yet. This study explores turning intersegmental coordination during smartphone use in different age groups and neurologic conditions.

**Objective:**

This study aims to evaluate the effect of smartphone use on turning behavior in healthy individuals of different ages and those with various neurological diseases.

**Methods:**

Younger (aged 18-60 years) and older (aged >60 years) healthy individuals and those with Parkinson disease, multiple sclerosis, subacute stroke (<4 weeks), or lower-back pain performed turning-while-walking alone (single task [ST]) and while performing 2 different cognitive tasks of increasing complexity (dual task [DT]). The mobility task consisted of walking up and down a 5-m walkway at self-selected speed, thus including 180° turns. Cognitive tasks consisted of a simple reaction time test (simple DT [SDT]) and a numerical Stroop test (complex DT [CDT]). General (turn duration and the number of steps while turning), segmental (peak angular velocity), and intersegmental turning parameters (intersegmental turning onset latency and maximum intersegmental angle) were extracted for head, sternum, and pelvis using a motion capture system and a turning detection algorithm.

**Results:**

In total, 121 participants were enrolled. All participants, irrespective of age and neurologic disease, showed a reduced intersegmental turning onset latency and a reduced maximum intersegmental angle of both pelvis and sternum relative to head, thus indicating an en bloc turning behavior when using a smartphone. With regard to change from the ST to turning when using a smartphone, participants with Parkinson disease reduced their peak angular velocity the most, which was significantly different from lower-back pain relative to the head (*P*<.01). Participants with stroke showed en bloc turning already without smartphone use.

**Conclusions:**

Smartphone use during turning-while-walking may lead to en bloc turning and thus increase fall risk across age and neurologic disease groups. This behavior is probably particularly dangerous for those groups with the most pronounced changes in turning parameters during smartphone use and the highest fall risk, such as individuals with Parkinson disease. Moreover, the experimental paradigm presented here might be useful in differentiating individuals with lower-back pain without and those with early or prodromal Parkinson disease. In individuals with subacute stroke, en bloc turning could represent a compensative strategy to overcome the newly occurring mobility deficit. Considering the ubiquitous smartphone use in daily life, this study should stimulate future studies in the area of fall risk and neurological and orthopedic diseases.

**Trial Registration:**

German Clinical Trials Register DRKS00022998; https://drks.de/search/en/trial/DRKS00022998

## Introduction

Turning-while-walking is relevant to everyday life and very common, accounting for >40% of steps taken in daily activities [1]. The movement itself requires a good coordination of body segments and control of body rotation toward the new direction while maintaining dynamic stability. In healthy adults, a top-down temporal sequence in body segments reorientation around the vertical axis, while turning has been described [2-5]. This starts with eyes and head movement to fix the gaze onto the new direction, followed by sternum, pelvis, and finally feet rotation. Being a challenging task, turning renders individuals at higher risk of falling, particularly in older adults and people who have mobility impairments such as neurologic patients [6,7].

Previous evidence supports the negative association of an effective top-down sequence of body segments reorientation with the risk of falls, with people reorientating body segments more simultaneously being more prone to have multiple falls [8]. This strategy is generally called en bloc turning. On these premises, some studies investigated the influence of kinematic and physiological factors and pathological conditions on turning behavior. An influence of kinematic factors such as turning velocity, on timing and magnitude of segmental reorientation, has been suggested; however, only a few studies have investigated these elements, with conflicting results [5,9,10]. Age also seems to have an influence on intersegmental turning behavior with the head, sternum, and pelvis turning more simultaneously in older subjects [9,11]. Considering the neurologic disorders, the results are often inconsistent with some studies showing a preserved top-down reorientation in chronic stroke [12-16] and individuals with Parkinson disease [17,18], while others studies reported en bloc turning behavior for these individuals [3,19-23]. This could depend, at least partially, on the different experimental protocols (eg, different turning magnitudes, on-the-spot turning, and turning-while-walking), enrolled populations, and measures used to investigate turning behavior (eg, number and selection of body segments, temporal and spatial measures). Only a few studies have investigated turning intersegmental coordination in individuals with lower-back pain [24,25] and focused only on the sternum and pelvis, while no studies, to the best of our knowledge, investigated turning intersegmental coordination in individuals with multiple sclerosis.

Turning is particularly dangerous when performed during multitasking. This term refers to the performance of multiple tasks simultaneously, such as walking and talking or driving while speaking at the phone. Multitasking is very common in everyday life, and several activities are performed while walking and turning. Performing a secondary task while walking (dual task [DT]) could negatively affect gait performance [26] and predict future falls [27], and several theories have been proposed to explain this effect, such as limited attention allocation capacity, a bottleneck effect for tasks performed in parallel, and competition for computational resources [28]. Differently from gait, only a few studies investigated the effect of DT on turning behavior. Most studies focused on general turning parameters such as the number of steps taken, turn duration, or peak angular velocity in both healthy subjects [29,30] and individuals with neurologic conditions [30-33]. Only 1 study, to the best of our knowledge, investigated the effect of DT on intersegmental coordination [23], showing a disruption of turning sequence in individuals who have chronic stroke only under cognitive DT condition, while healthy participants showed a preserved top-down sequence.

Similarly, no study to date has investigated the effect of smartphone use on turning behavior. This is a ubiquitous condition in everyday life, and in the last decade, some evidence has shown a detrimental effect of smartphone use on straight walking and balance [34-37].

To our best knowledge, this is the first study evaluating the effect of smartphone use on turning behavior in healthy participants of different ages and those with various neurological diseases.

## Methods

### Population

Participants were recruited through flyers placed in public facilities (healthy participants) and in the Department of Neurology and outpatient clinics at University Hospital Schleswig-Holstein, Campus Kiel, Germany (neurological patients). Inclusion criteria were (1) being aged 18 years and older and (2) ability to walk independently without walking aids. Exclusion criteria were (1) a Montreal Cognitive Assessment (MoCA) score of <15 and (2) other movement impairments affecting mobility performance, as judged by the assessor. Participants were divided into 6 groups according to age and diagnosis. Healthy participants were divided into “young” (aged 18-60 years) and “older” (aged > 60 years). Participants with neurological disorders included individuals with Parkinson disease (according to the UK Brain Bank criteria [38]), multiple sclerosis (according to McDonalds criteria [39]), subacute stroke (<4 weeks), or lower-back pain as indicated on the basis of the patients’ medical history and diagnosis upon examination [40].

### Ethics Approval

The study was approved by the ethical committee of the Medical Faculty of Kiel University (D438/18) and was conducted in accordance with the principles of the Declaration of Helsinki. All participants provided written informed consent before the start of measurements. The study is registered in the German Clinical Trials Register (DRKS00022998, registration date: September 4, 2020).

### Demographic and Clinical Data

Demographic data including age, sex, weight, and height were collected. Overall cognitive function at baseline was assessed with the MoCA [41]. Participants' mobility was assessed with the Short Physical Performance Battery [42]. Disease-specific evaluation included the Movement Disorder Society Unified Parkinson's Disease Rating Scale part III [43] and the Hoehn and Yahr Scale [44] for participants with Parkinson disease (performed in ON condition); the Expanded Disability Status Scale [45] for participants with multiple sclerosis; the National Institute of Health Stroke Scale [46] for participants with subacute stroke, and a visual analog scale of pain intensity [47], and the German Funktionsfragenbogen Hannover Scale [48] for participants with lower-back pain.

### Experimental Procedure

**Figure 1:**
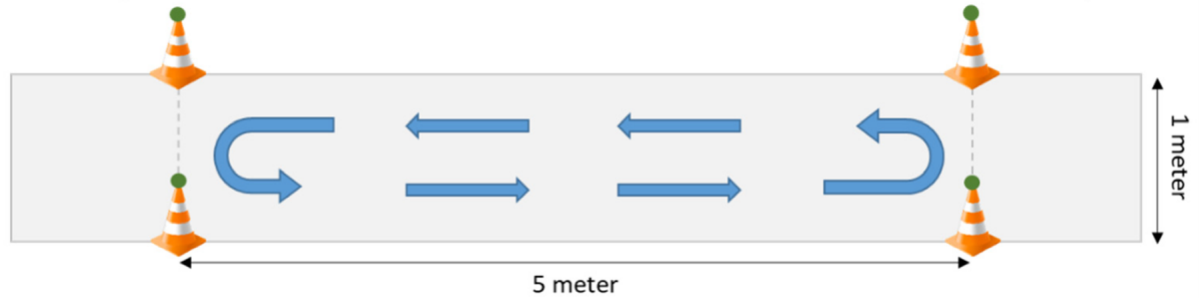
Schematic representation of the 5-m walkway and the turning-while walking task.

Participants were asked to perform an overground walking and turning task on a 5-m-long walkway with a width of 1 m. Participants were asked to walk up and down the walkway at self-selected speed, thus including 180° turns to change walking direction (Figure 1). Walking and turning tasks were performed as a single task (ST) for a duration of 30 seconds, and while performing 2 different cognitive tasks of similar duration (~30 seconds) of increasing complexity: a simple reaction time test (simple DT [SDT]) and a numerical Stroop test (complex DT [CDT]). Both cognitive tasks were administered via a handheld smartphone. ST, SDT, and CDT conditions were performed consecutively. In SDT, participants had to tap the screen as fast as possible after the appearance of a black square. In CDT, participants had to choose the higher number from 2 options [49]. The Stroop test was administered under 3 conditions: (1) congruent, in which the number with a higher value had a larger character size; (2) neutral, in which the character size of both numbers was equal; (3) incongruent, in which the number with a higher value had a smaller character size. Participants performed a practice trial on both ST and DT conditions to familiarize themselves with the procedure. More details on the study protocol can be found elsewhere [50].

### Movement and Rotation Analysis

A 12-camera 3D optical motion capture system (Qualisys AB) was used to record marker trajectories of passive retroreflective markers attached on the skin or tight clothing and footwear of the participants. Reflective markers were placed, among others, on the head, sternum, pelvis, and left and right feet. The exact placement of the markers is described elsewhere [50]. Data were recorded at 200 Hz. The orientation of the head, sternum, and pelvis was calculated based on the reflective markers on each of these segments. To detect turns during walking, angles of the head, sternum, and pelvis were calculated according to Euler method using the orientation of the segments derived from the position of the reflective markers [51]. The start and end of a turn were detected by the change in SD. A change in SD from the mean indicates an abrupt change in the signal. At the start and end of a turn, an abrupt change in the angular signal around the vertical axis can be seen. The detection of this abrupt change in signal was carried out with the MATLAB function *findchangepts*. This function detects the point at which a mean changes most significantly by partitioning data into 2 regions that minimize the sum of the residual (squared) error of each region from its local mean. Steps were detected from the heel and toe marker trajectories. For this purpose, vertical velocity signals were computed from the heel and toe markers’ position data [52], and initial contact was determined from a local minimum in the vertical velocity of the toe marker, whereas final contact was determined from a local maximum in the vertical velocity of the heel marker [53].

### Measures

For the analysis of turning behavior, we calculated general turning parameters as well as segmental and intersegmental measures for the head, sternum, and pelvis. Regarding general parameters, turn duration, defined as the time in seconds between the beginning and ending of the turning phase, and the number of steps taken while turning were calculated. Concerning segmental turning measures, the peak angular velocity for each of the 3 body segments was measured. Finally, to investigate intersegmental coordination during turning in the temporal and spatial domains, we calculated intersegmental relative turning onset latencies and intersegmental maximum angles, respectively. These were calculated for each pair of segments for a total of 3 pairs. Turning onset latencies were determined using the more cranial one as a reference in each pair (sternum relative to the head, pelvis relative to the head, and pelvis relative to the sternum). A negative value indicates that the cranial segment started turning first. For each participant, variables were calculated as the average across turns for each of the 3 conditions.

### Data and Statistical Analysis

To evaluate the impact of DT on turn duration, number of steps, and peak angular velocity for the 3 body segments, dual-task cost (DTC) was calculated for both SDT and CDT on the basis of equation 1 [54,55]:



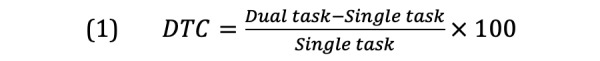



Statistical analyses were performed using JASP (version 0.16.1; JASP Team), R (version 4.0.3; The R Foundation), and RStudio (version 2022.02.2+433 for Windows; R Foundation for Statistical Computing). Descriptive statistics were calculated for the examined variables. To assess the difference in turn duration, number of steps, peak angular velocity, turning onset latencies, and maximum angles across the different groups and different conditions, mixed ANOVAs, corrected for gait speed, were used, with the factors “condition” and “group.” Post hoc *t* tests with Bonferroni correction were performed in case of significant ANOVA main effects. To assess the difference in DTC for turn duration, number of steps, and peak angular velocity between the different groups and between SDT and CDT, mixed ANOVAs, corrected for gait speed, were used, with the factors “DT condition” and “group.” To evaluate the segmental sequence of body segment while turning, mixed ANOVAs, corrected for gait speed, with the factors “segment pair turning onset latency” and “group” were used. If necessary, Greenhouse-Geisser correction for nonsphericity were applied. The significance threshold was set at α<.05. All data were reported as mean (SD) or median (IQR) for numerical data and N (%) for categorical variables.

## Results

**Figure 2:**
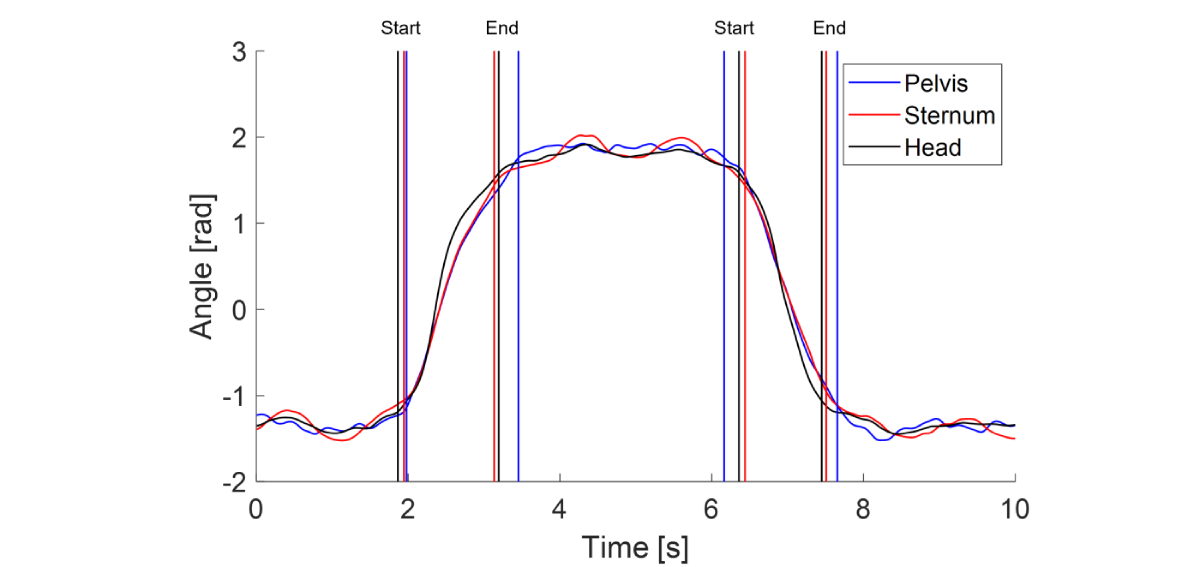
Example of the turning detection algorithm output. Angular signals of the pelvis, sternum, and head are reported. The vertical lines indicate the start and end of a turn for each body segment.

A total of 121 participants were enrolled in the study. Table 1 shows the demographic and clinical data of the included groups. Average turn magnitudes detected by our algorithm for head, sternum, and pelvis were 160° (SD 8°), 153° (SD 7°), and 155° (SD 7°), respectively. An example of algorithm output is shown in Figure 2.

**Table 1:** Demographics and clinical data of the enrolled groups.

			Young adults (N=36)		Older adults (N=18)		Participants with Parkinson disease (N=26)		Participants with subacute stroke (N=14)		Participants with multiple sclerosis (N=19)		Participants with lower-back pain (N=8)
Age (years), mean (SD)			28.9 (8.3)		71.9 (6.3)		63.3 (10.9)		63.7 (16.6)		38.5 (13.0)		63.6 (16.8)
**Sex, n (%)**													
	Male	15 (42)		9 (50)		10 (38)		3 (21)		11 (58)		3 (37)	
	Female	21 (58)		9 (50)		16 (62)		11 (79)		8 (42)		5 (63)	
Height (cm), mean (SD)			180 (9)		173 (10)		175 (8)		175 (11)		179 (11)		174 (10)
Weight (kg), mean (SD)			74.8 (14)		78.4 (16.3)		83.4 (15.2)		81.4 (16.5)		81.7 (21.2)		79.8 (18.1)
BMI, mean (SD)			22.9 (3)		26.1 (5.1)		27.1 (4)		26.4 (4.8)		25.3 (4.7)		26.3 (3.6)
MoCA^a^ score, median (IQR)			29 (28-30)		23.5 (21-27)		25 (23-26)		23 (20-26)		28 (26-29)		25 (24-27)
SPPB^b^ score, median (IQR)			12 (12-12)		11 (9-11)		10 (8-11)		11 (9-11)		9 (7-11)		12 (8-12)
H&Y^c^ score, median (IQR)			—^d^		—		2 (1-3)		—		—		—
MDS-UPDRS-III^e^ score, median (IQR)			—		—		22 (12-28)		—		—		—
EDSS^f^ score, median (IQR)			—		—		—		—		1 (1-4)		—
NIHSS^g^ score, median (IQR)			—		—		—		0 (0-3)		—		—
pVAS^h^ score, median (IQR)			—		—		3 (0-6)		—		—		4 (1-6)
FFbH^i^ score, median (IQR)			—		—		30 (27-32)		—		—		3 (29-31)

^a^MoCA: Montreal Cognitive Assessment.

^b^SPPB: Short Physical Performance Battery.

^c^H&Y: Hoehn and Yahr Scale.

^d^Not determined.

^e^MDS-UPDRS-III: Movement Disorder Society Unified Parkinson's Disease Rating Scale part III.

^f^EDSS: Expanded Disability Status Scale.

^g^NIHSS: National Institute of Health Stroke Scale.

^h^pVAS: visual analog scale of pain intensity.

^i^FFbH: Funktionsfragenbogen Hannover Scale.

### Turning Sequence

Significant effects of factor “group” (*F*_5,99_=2.689; *P=*.03) and “segment pair turning onset latency” (*F*_1.51,149.03_=104.5; *P<*.001) but no significant interaction between the 2 factors were found in ST condition. No significant effects of the 2 factors, or interaction between the 2 factors, were found for the SDT or the CDT condition. Considering factor “segment pair,” post hoc analysis showed a significant difference between turning onset latency of the pelvis relative to the sternum and both turning onset latency of the sternum relative to the head and the pelvis relative to the head in all groups (all *P<*.01), except in participants with subacute stroke.

### Turn Duration and Number of Steps

Significant effects of factor “group” and “condition,” but no significant interaction between the 2 factors were found for turn duration (*F*_5,83_=8.091, *P<*.001; *F*_1.40,116.42_=18.269, *P<*.001) and number of steps while turning (*F*_5,78_=2.988, *P=*.02; *F*_1.45,112.79_=12.4, *P<*.001).

### Peak Angular Velocity

Significant effects of factor “group” and “condition” were found for peak angular velocity of the head (*F*_5,99_=12.878, *P<*.001; *F*_1.57,155.86_=112.506, *P<*.001), sternum (*F*_5,95_=8.569, *P<*.001; *F*_1.51,146.67_=4.204, *P=*.03), and pelvis (*F*_5,94_=10.171, *P<*.001; *F*_1.76,165.47_=24.844, *P<*.001). A significant interaction between the 2 factors was found only for peak angular velocity of the pelvis (*F*_8.80,165.47_=2.520; *P=*.01).

### Intersegmental Relative Turning Onset Latencies

**Figure 3:**
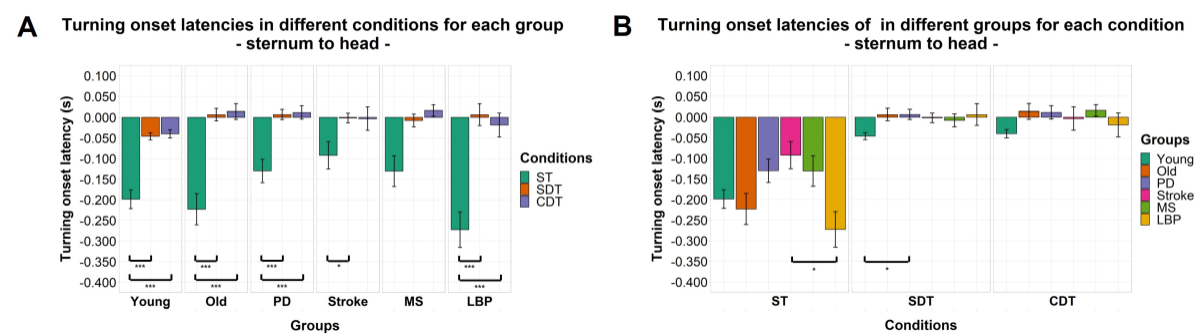
Turning onset latencies by group (A) and task condition (B) of the sternum relative to the head. Mean of the variables is represented by bar height. SE of the mean is shown by the vertical lines. A negative value means that the cranial body segment starts turning first and vice versa. Significant pairwise comparisons are marked by horizontal lines and asterisks as follows: *P<.05; **P<.01; ***P<.001. CDT: complex dual task; LBP: Lower-back pain; MS: multiple sclerosis; PD: Parkinson disease; SDT: simple dual task; ST: single task.

**Figure 4:**
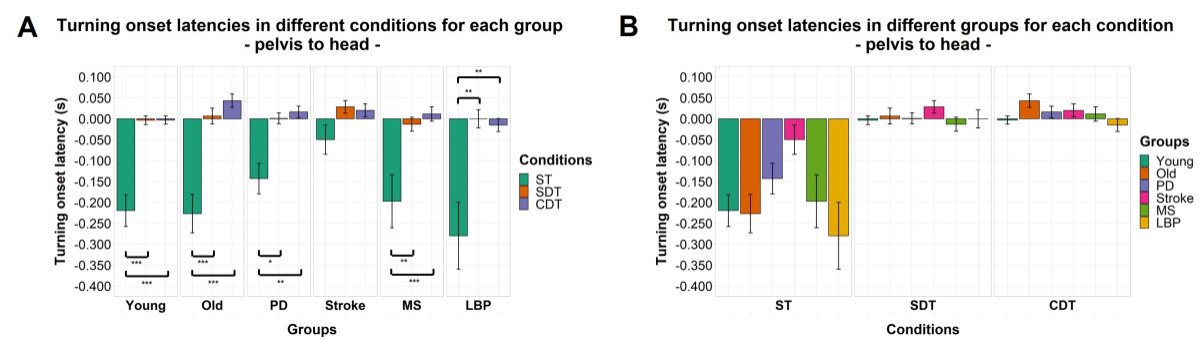
Turning onset latencies by group (A) and task condition (B) of the pelvis relative to the head. Mean of the variables is represented by bar height. SE of the mean is shown by the vertical lines. A negative value means that the cranial body segment starts turning first and vice-versa. Significant pairwise comparisons are marked by horizontal lines and asterisks as follows: *P<.05; **P<.01; ***P<.001. CDT: complex dual task; LBP: Lower-back pain; MS: multiple sclerosis; PD: Parkinson disease; SDT: simple dual task; ST: single task.

**Figure 5:**
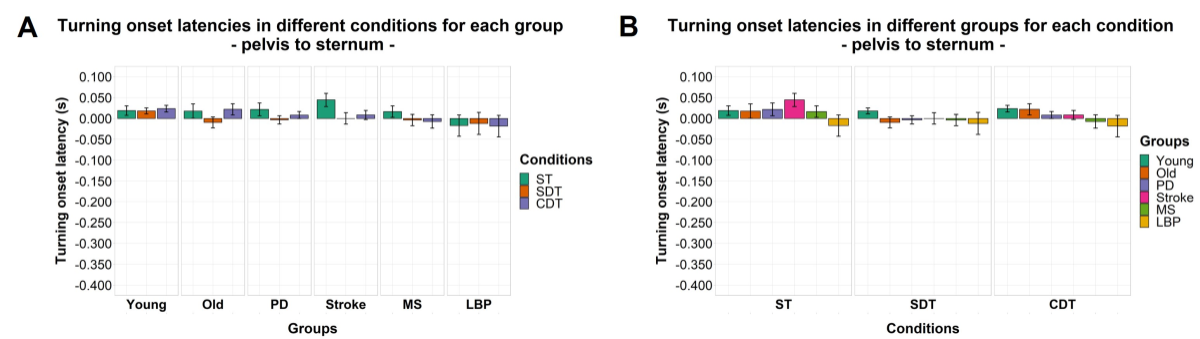
Turning onset latencies by group (A) and task condition (B) of the pelvis relative to the sternum. Mean of the variables is represented by bar height. SE of the mean is shown by the vertical lines. A negative value means that the cranial body segment starts turning first and vice versa. Significant pairwise comparisons are marked by horizontal lines and asterisks as follows: *P<.05; **P<.01; ***P<.001. CDT: complex dual task; LBP: Lower-back pain; MS: multiple sclerosis; PD: Parkinson disease; SDT: simple dual task; ST: single task.

Significant effects of factor “condition” were found for both turning onset latency of the sternum relative to the head (*F*_1.39,120.82_=116.121; *P<*.001) and the pelvis relative to the head (*F*_1.15,99.75_=66.019; *P<*.001). A significant effect of factor “group” and interaction between the 2 factors was found only for turning onset latency of the sternum relative to the head (*F*_5,87_=2.683, *P=*.03; *F*_6.94,120.82_=2.109; *P=*.048). No significant effects or interaction between the 2 factors were found for turning onset latency of the pelvis relative to the sternum. Details of post hoc comparisons can be found in Figures 3-5.

### Intersegmental Maximum Angles

**Figure 6:**
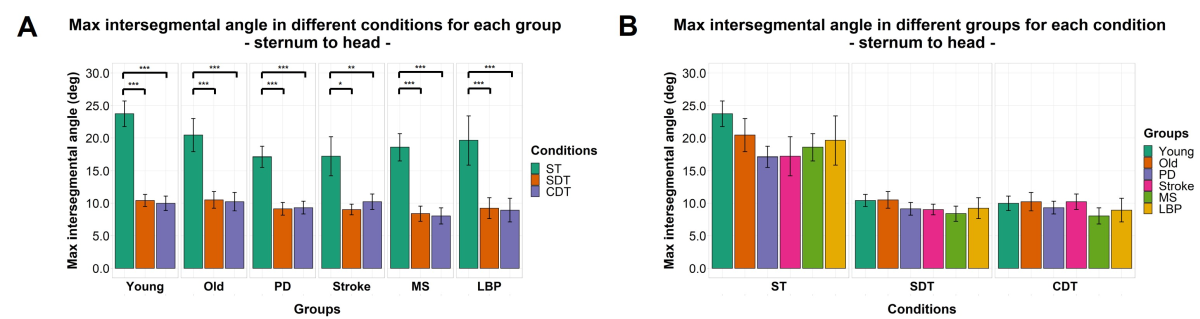
Maximum intersegmental angles by group (A) and task condition (B) between sternum and head. Mean of the variables is represented by bar height. SE of the mean is shown by the vertical lines. Significant pairwise comparisons are marked by horizontal lines and asterisks as follows: *P<.05; **P<.01; ***P<.001. CDT: complex dual task; LBP: Lower-back pain; MS: multiple sclerosis; PD: Parkinson disease; SDT: simple dual task; ST: single task.

**Figure 7:**
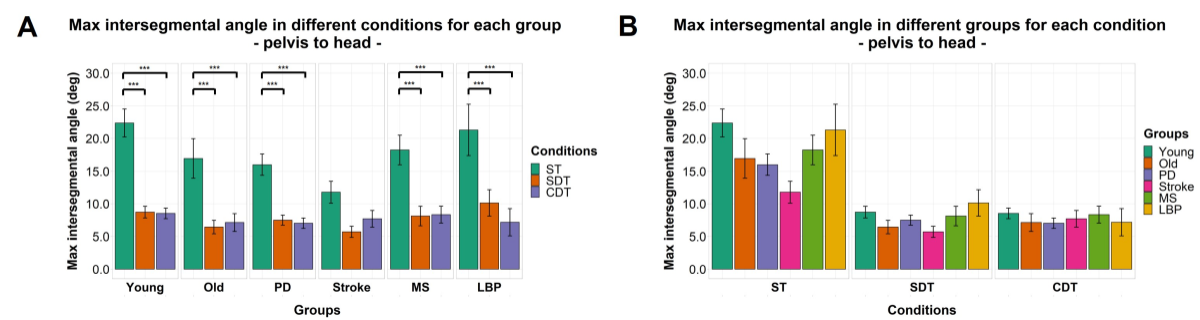
Maximum intersegmental angles by group (A) and task condition (B) between pelvis and head. Mean of the variables is represented by bar height. SE of the mean is shown by the vertical lines. Significant pairwise comparisons are marked by horizontal lines and asterisks as follows: *P<.05; **P<.01; ***P<.001.CDT: complex dual task; LBP: Lower-back pain; MS: multiple sclerosis; PD: Parkinson disease; SDT: simple dual task; ST: single task.

**Figure 8:**
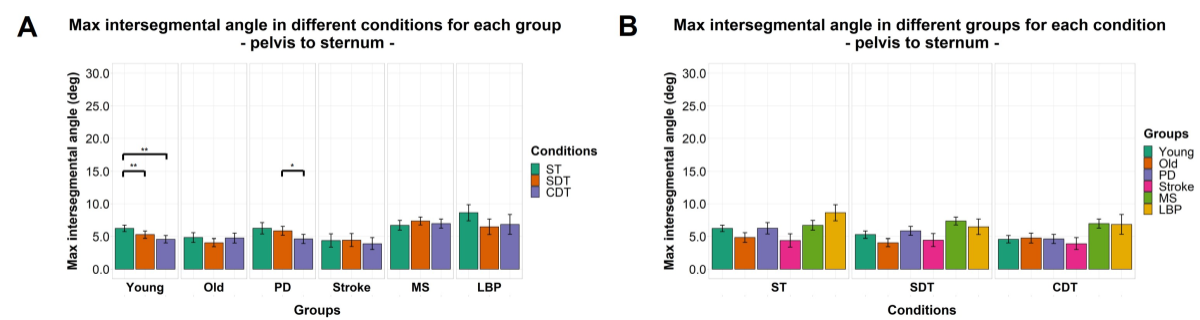
Maximum intersegmental angles by group (A) and task condition (B) between pelvis and sternum. The mean of the variables is represented by bar height. SE of the mean is shown by the vertical lines. Significant pairwise comparisons are marked by horizontal lines and asterisks as follows: *P<.05; **P<.01; ***P<.001. CDT: complex dual task; LBP: Lower-back pain; MS: multiple sclerosis; PD: Parkinson disease; SDT: simple dual task; ST: single task.

Significant effects of factor “condition” were found for maximum angle between the head and sternum (*F*_1.30,128.97_=104.478; *P<*.001), head and pelvis (*F*_1.24,115.65_=93.328; *P<*.001), and the sternum and pelvis (*F*_1.73,164.21_=4.660; *P=*.01) but not for factor “group” or interaction between the 2 factors for each of the 3 segments pairs. Details of post hoc comparisons can be found in Figures 6-8.

### Dual-Task Cost

**Figure 9:**
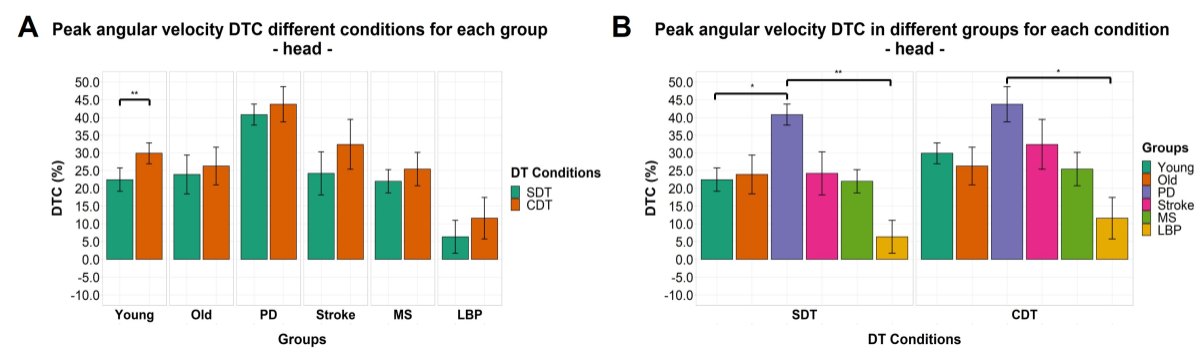
Dual task cost of peak angular velocity of the head by group (A) and task condition (B). The mean of the variables is represented by bar height. SE of the mean is shown by the vertical lines. Significant pairwise comparisons are marked by horizontal lines and asterisks as follows: *P<.05; **P<.01; ***P<.001. CDT: complex dual task; DT: dual task; DTC: dual-task cost; LBP: Lower-back pain; MS: multiple sclerosis; PD: Parkinson disease; SDT: simple dual task; ST: single task.

A significant effect of factor “DT condition” (*F*_1,70_=7.216; *P=*.009) but not for factor “group” or interaction between the 2 factors was found for turn duration DTC. No significant effects or interactions between the 2 factors were found for DTC of number of steps while turning. Significant effects of factor “group” were found for DTC of peak angular velocity for head (*F*_5,91_=3.701; *P=*.004) and sternum (*F*_5,72_=3.2.382; *P=*.047). A significant effect of factor “DT condition” was found for DTC of peak angular velocity for the head (*F*_5,91_=7.028; *P=*.009) and pelvis (*F*_1,82_=8.237; *P=*.005). No interaction between the 2 factors was found for all 3 body segments. Post hoc analysis showed a significant difference between participants with Parkinson disease and those with lower-back pain for DTC of peak angular velocity for the head in both SDT and CDT conditions with participants with Parkinson disease, demonstrating a higher DTC (mean difference 33%, SD 9% and 32%, SD 10%, respectively; Figure 9).

More details regarding the pairwise comparisons for the investigated variables can be found in the Multimedia Appendix 1.

## Discussion

### Overview

This cross-sectional study aimed at evaluating the effects of concurrent active smartphone usage and turning-while-walking on the dynamics of intersegmental coordination of turning. Both turning and smartphone use during walking are common in human life, and reduced turning coordination could lead to an increased risk of falling [8,56]. Smartphone use has already been associated with poorer balance and gait [35-37], but no study to date investigated the effect of smartphone use on turning behavior. Moreover, advanced age as well as several neurologic conditions are known to be associated with an increased risk of falling, and smartphone usage during turning might be particularly critical for specific groups. To determine the effects of age and neurologic conditions, both young and older participants as well as those with different neurologic disorders were included into the study.

### Principal Results

We found that all participants, irrespective of age and neurologic disease, showed an en bloc turning behavior when using a smartphone; participants with Parkinson disease showed the most pronounced reduction in peak angular velocity, with a significant difference for the DTC of head segment compared to lower-back pain; and that our participants with subacute stroke turned en bloc even without a smartphone. These results are discussed in detail in the following paragraphs.

All groups turned en bloc while performing a cognitive task on a smartphone irrespective of age and neurologic condition. Although the direct effect of smartphone use on tuning has not been explored to date, reports investigating the influence of smartphone on straight walking and balance showed a reduction in gait speed [35,36,57], and altered dynamic and static balance [37]. This was linked to the cognitive load of smartphone use but also to the need to reduce and smoothen head movements to maintain gaze fixation onto the screen [57]. The increased turn duration and the reduction of head peak angular velocity, while using a smartphone in this study is, at least indirectly, in line with these previous findings. Importantly, impaired turning coordination and en bloc turning have been associated with the fear of falling and higher risk of falls [8,56]. Therefore, the result that all participants, irrespective of disease and age, and even young healthy adults, turn en bloc while using a smartphone could have an important impact on future recommendations for smartphone use while walking.

We found that participants with Parkinson disease showed the lowest head peak angular velocity in all conditions. They also showed the longest turn duration and needed most steps for the turns. This was an expected result considering that bradykinesia and rigidity as well as reduced gait speed and increased cadence with shorter step length are typical features of Parkinson disease [58,59]. Interestingly, participants with Parkinson disease also showed the most marked reduction in peak angular velocity of all 3 investigated body segments when using a smartphone during turning, reflected by the highest peak angular velocity DTC. The latter observation is, in our view, surprising and difficult to explain by the mere presence of bradykinesia and rigidity. We assume that this marked reduction in peak angular velocity is more of a cognitive phenomenon. Individuals with Parkinson disease have been reported to be particularly prone to experience DT deficits, particularly during walking [27,60]. Compared to walking, turning is a more complex and demanding task, and the simultaneous “triple task” (ie, smartphone use, turning, and cognitive task) could represent a bottleneck in neural processing that could lead to dangerous turning behavior, especially in individuals with Parkinson disease. For people with Parkinson disease experiencing falls, it could therefore be useful to consider that turning while using a smartphone constitutes a particular fall risk, and then to work out whether the risk is influenced more by the motor component, the cognitive component or by a combination of both.

Participants with lower-back pain were the only group that did not show any significant difference in head peak angular velocity when using a smartphone, compared with ST. This was particularly interesting due to the following observation. Although participants with Parkinson disease and those with lower-back pain were comparable concerning demographic and clinical (including pain intensity) parameters, both groups were remarkably different when comparing head peak angular velocity DTC (AUC of 0.96 and 0.92 for SDT and CDT, respectively, see also Multimedia Appendix 1). Considering that lower-back pain and similar pain conditions are common in prodromal [61,62] and clinically evident Parkinson disease [63,64], we speculate that the experimental paradigm presented here could be useful in differentiating individuals with lower-back pain without and those with (prodromal) Parkinson disease and future specifically designed studies could help confirming this hypothesis.

In our participants with subacute stroke, despite the head started turning slightly before sternum and pelvis, there was no significant difference in turning onset latencies among the 3 segment pairs even in the turning-while-walking–only condition. We hypothesize that en bloc turning may not be deleterious under all circumstances but, for example, in acute or subacute medical situations, may even serve as a compensative strategy to overcome the newly occurring mobility deficit. Increased cocontraction and impedance control with the goal of reducing kinematic errors, stabilizing movement, and increasing performance is a common strategy used in the early phases of motor learning when new dynamics have to be acquired [65]. Differing results from previous studies investigating intersegmental turning in individuals with stroke may, at least indirectly, also argue in this direction considering that all previous studies [12-16] included individuals with chronic stroke. Therefore, our results could pave the way for new rehabilitation strategies targeting gait and turning in individuals with subacute stroke.

### Limitations

This study faces some limitations. First, the sample size at least of some groups, the range of disease severity, and the generally relatively high level of physical and cognitive abilities of our participants may limit the generalizability of our results, and further studies including participants with lower functional scores, greater disease severity, and more severe cognitive impairment may be helpful to address this. Second, in this proof-of-concept study, we chose a 180° turn paradigm. However, we are aware that other, primarily smaller turns are also performed in everyday life, and future studies should investigate the influence of smartphone use on these turns. Third, on average, the turning algorithm used in this study underestimated the turning magnitudes by 10%-15%. This could likely be attributed to the general structure of the algorithm (see the *Methods* section) in combination with the ellipse-like, “nonabrupt” turning trajectory. As this is a systematic bias observed in all experimental conditions and in all body segments, we assume that it is an irrelevant aspect of our data presented here, but future studies may use additional or alternative turning algorithms for data analysis. Finally, we included only smartphone tasks in which subjects had to interact with the touchscreen and did not include tasks in which participants had to walk while talking on the phone. The latter is also a very common type of smartphone use, and should therefore also be investigated concerning influence on turning behavior in future studies.

### Conclusions

Performing a secondary task on a smartphone leads to a more en bloc turning irrespective of age and neurologic condition. The segmental turning behavior of participants with Parkinson disease suggests that this disease could be most affected by smartphone use and these participants could be at high risk of falling when turning while using a smartphone. Considering the ubiquitous smartphone use in daily life, results of this study could stimulate future studies in this area, as well as, in clinical routine, the type of history taken from elderly and neurologically ill individuals who have increased risk of falling during ambulation.

## Appendix

Multimedia Appendix 1 Supplementary figures and tables.

## Abbreviations

CDT: complex dual task
DT: dual task
DTC: dual-task cost
MoCA: Montreal Cognitive Assessment
SDT: simple dual task
ST: single task

## Data Availability

Data of a subgroup of healthy participants included in our study are published digitally [66] and are made available using Figshare. The rest of the data presented in this study are available on request from the corresponding author.
