# Supplementary material for: Turning When Using Smartphone in Persons With and Those Without Neurologic Conditions: Observational Study
Source: J Med Internet Res. 2023 Mar 30;25:e41082. doi: 10.2196/41082 (PMC10131647; doi:10.2196/41082)
Supplement: Multimedia Appendix 1 [file jmir_v25i1e41082_app1.docx]

**Supplementary Figure 1. Turning duration by group and task condition.** Panel A and C: Turn duration in seconds (A) and number of steps taken while turning (C) across the different conditions in each group. Panel B and D: Turn duration (B) and number of steps taken while turning (D) across the different groups in each condition. Mean of the variables is represented by bar height. SE of the mean is shown by the vertical lines. Significant pairwise comparisons are marked by horizontal lines and asterisks as follows: * = significant for α < 0.05; ** = significant for α < 0.01; *** = significant for α < 0.001. ST: Single Task; SDT: Simple Dual Task; CDT: Complex Dual Task.


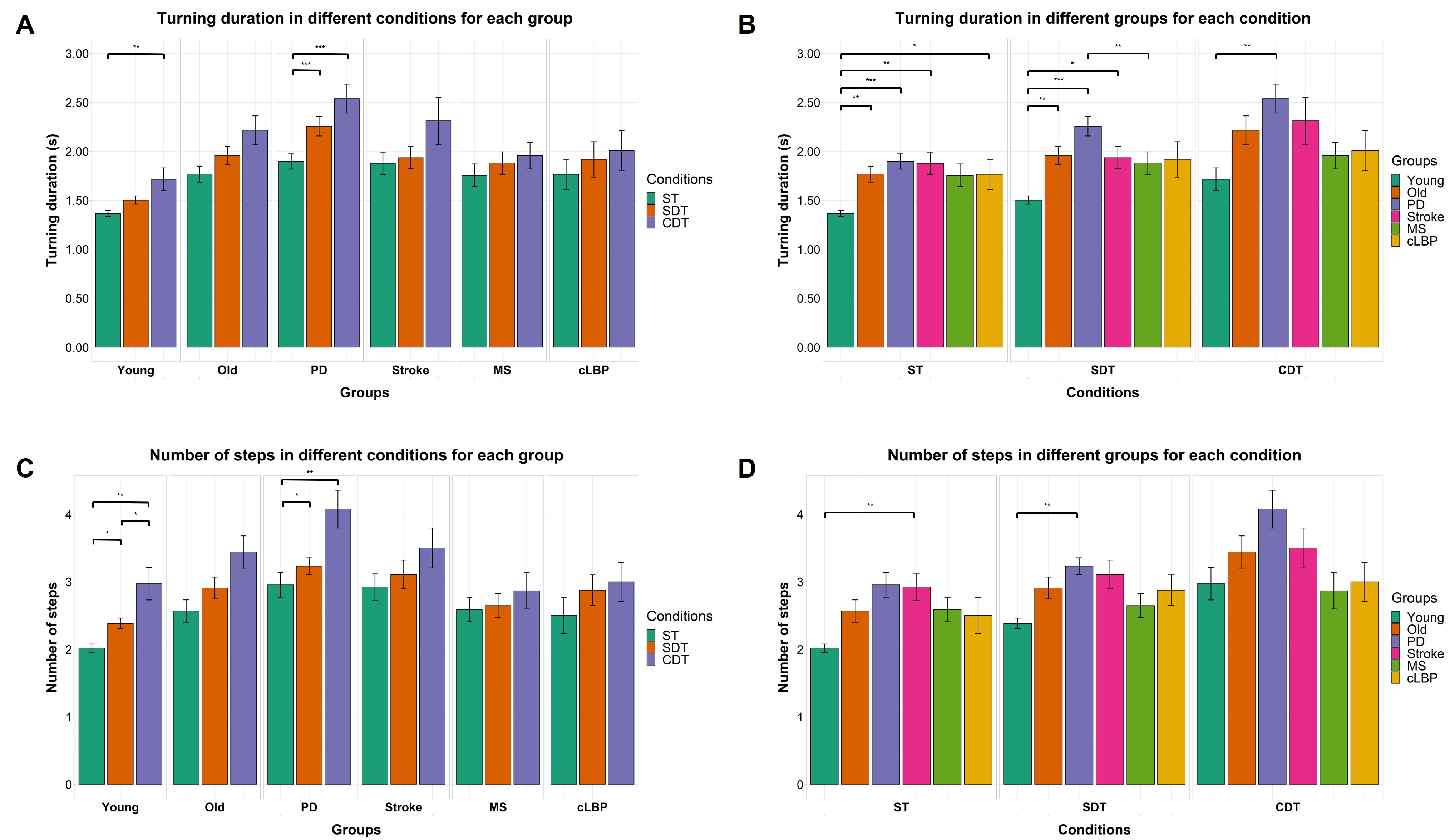


**Supplementary Figure 2. Dual task cost of turn duration and number of steps in healthy individuals and patients with neurologic conditions.** Panel A and C: DTC of turn duration (A) and number of steps taken while turning (C) between the DT conditions in each group. Panel B and D: DTC of turn duration (B) and number of steps taken while turning (D) across the groups in the two DT conditions. Mean of the variables is represented by bar height. SE of the mean is shown by the vertical lines. Significant pairwise comparisons are marked by horizontal lines and asterisks as follows: * = significant for α < 0.05; ** = significant for α < 0.01; *** = significant for α < 0.001. DTC: Dual Task Cost; SDT: Simple Dual Task; CDT: Complex Dual Task.

**
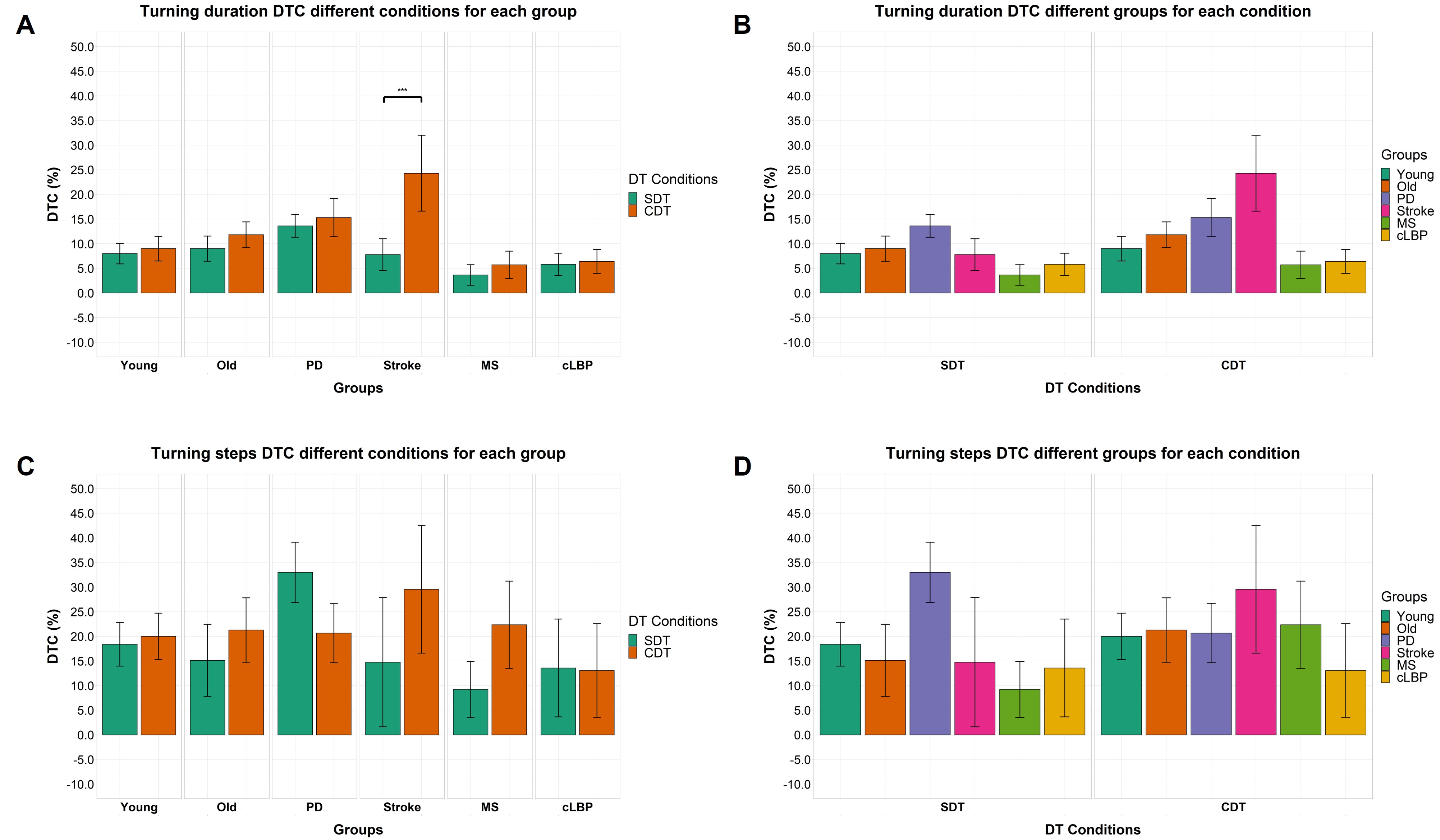
**

**Supplementary Figure 3. Peak angular velocity by segment, group and task condition.** Panel A, C and E: Peak angular velocity in degree/s of head (A), sternum (C) and pelvis (E) across the different conditions in each group. Panel B, D and F: Peak angular velocity in degree/s of head (B), sternum (D) and pelvis (F) across the groups in each condition. Mean of the variables is represented by bar height. SE of the mean is shown by the vertical lines. Significant pairwise comparisons are marked by horizontal lines and asterisks as follows: * = significant for α < 0.05; ** = significant for α < 0.01; *** = significant for α < 0.001. ST: Single Task; SDT: Simple Dual Task; CDT: Complex Dual Task.

**
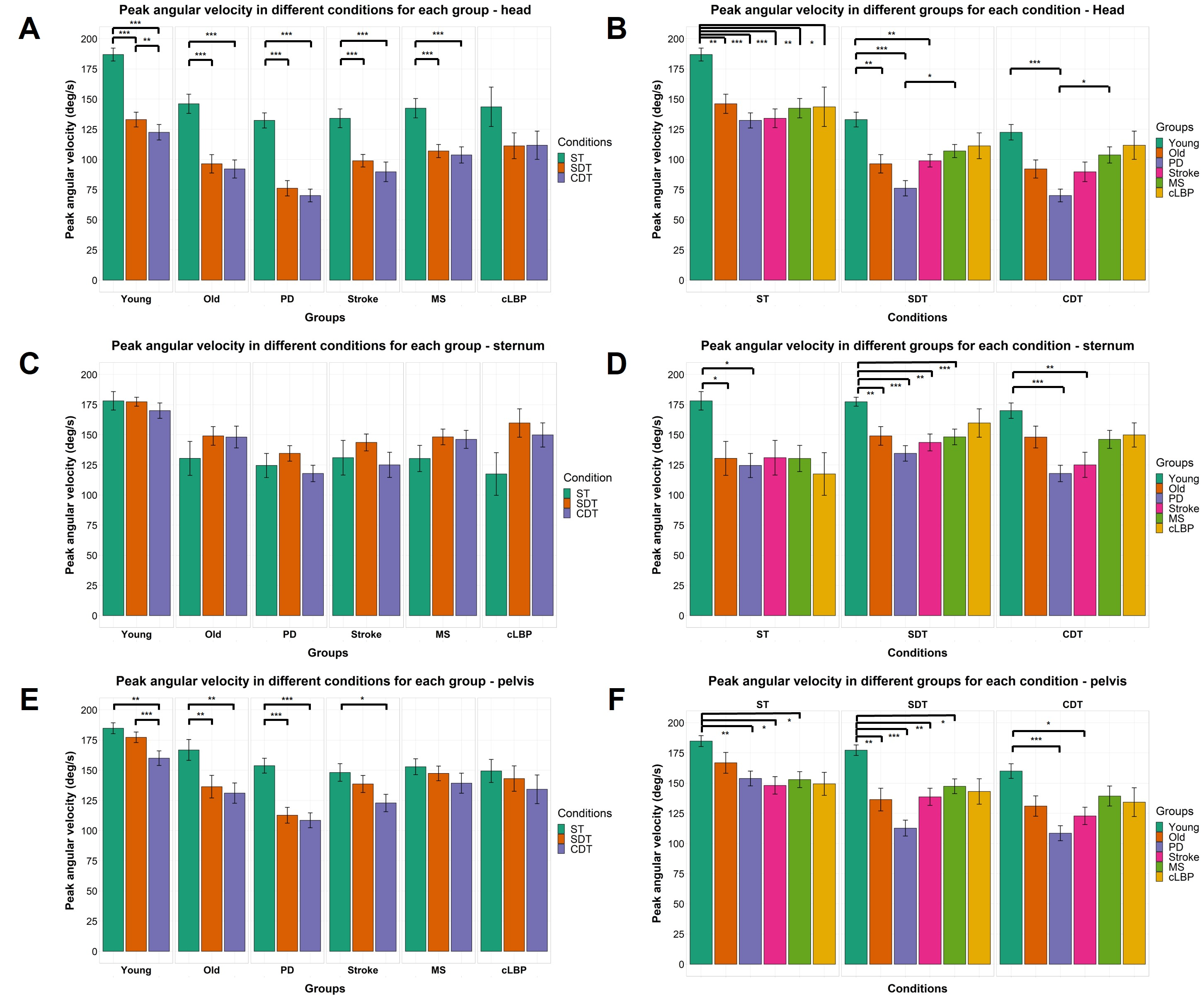
**

**Supplementary Figure 4. Dual task costs of peak angular velocity by segment, group, and task condition.** Panel A and C: DTC of peak angular velocity of sternum (A) and pelvis (C) between the two DT conditions in each group. Panel B and D: DTC of peak angular velocity of sternum (B) and pelvis (D) across the groups in the two DT conditions. Mean of the variables is represented by bar height. SE of the mean is shown by the vertical lines. Significant pairwise comparisons are marked by horizontal lines and asterisks as follows: * = significant for α < 0.05; ** = significant for α < 0.01; *** = significant for α < 0.001. DTC: Dual Task Cost; SDT: Simple Dual Task; CDT: Complex Dual Task.

**
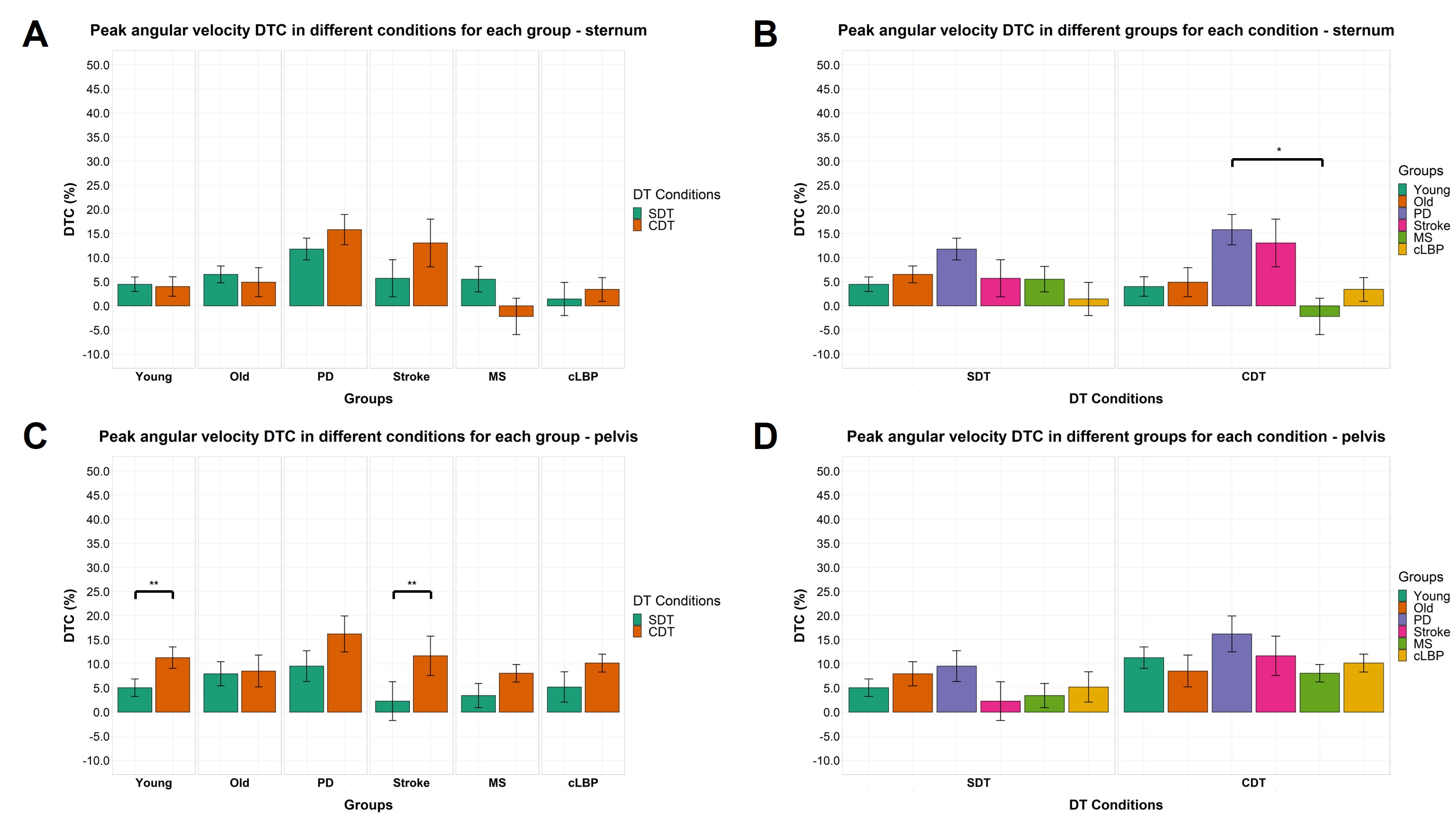
**

**Supplementary Figure 5. Receiver Operating Characteristic (ROC) curve for DTC of peak angular velocity of head in differentiating PD and LBP patients.** Panel A: ROC curve under SDT condition; Panel B ROC curve under CDT condition. CDT: Complex Dual-Task; DTC: Dual-Task cost; LBP: Lower-back Pain PD: Parkinson’s Disease; ROC: Receiver Operating Characteristic; SDT: Simple Dual-Task.

**
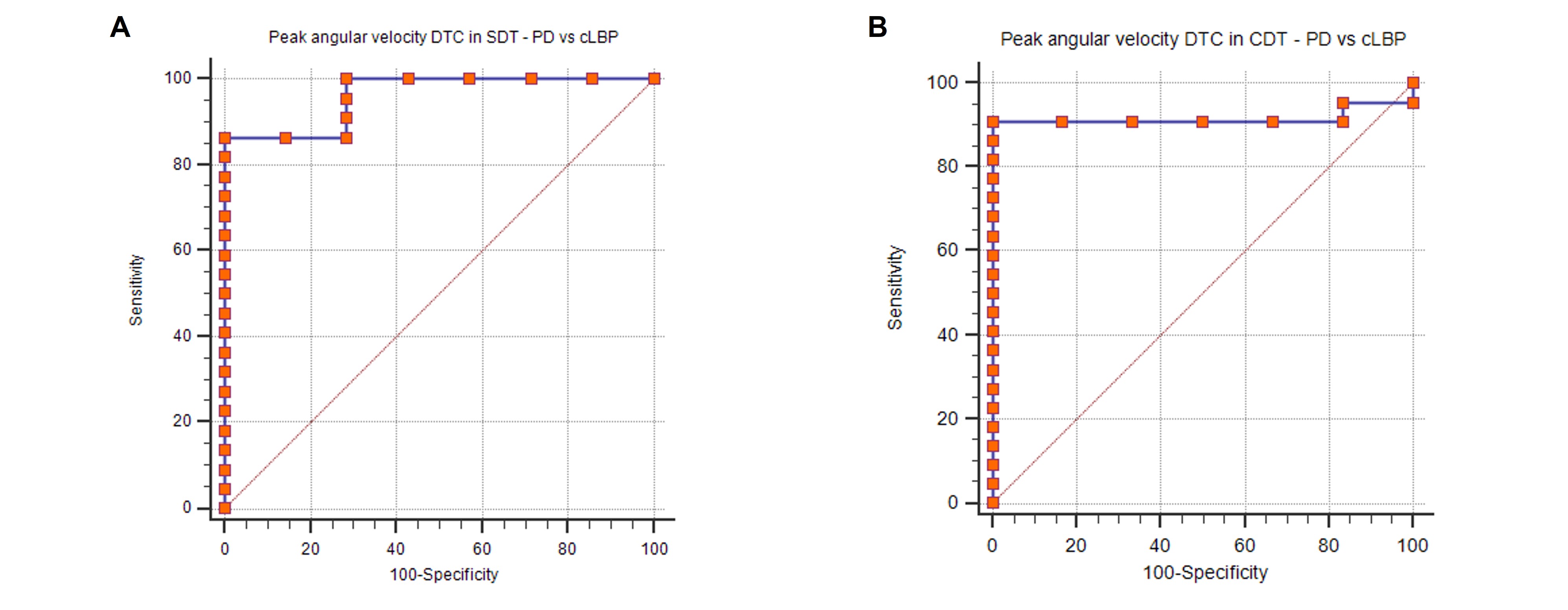
**

**Supplementary table 1.** ROC analysis for DTC of peak angular velocity of head in differentiating PD and LBP patients.

|  | SDT | CDT |
| --- | --- | --- |
| AUC | 0.961 | 0.917 |
| AUC SE | 0.033 | 0.058 |
| AUC 95% CI | 0.816 – 0.999 | 0.749 – 0.987 |
| AUC z statistic | 13.963 | 7.152 |
| Significance level (p) | <0.001 | <0.001 |
| YI | 0.864 | 0.909 |
| YI 95% CI | 0.682 – 1.000 | 0.727 – 1.000 |
| YI-associated criterion | >25.830 | >29.836 |
| YI-associated criterion 95% CI | 20.467 – 25.830 | 26.997 – 29.837 |

AUC: Area Under the Curve; CDT: Complex Dual-Task; CI: Confidence Interval; DTC: Dual Task Cost; ROC: Receiver Operating Characteristic; SDT: Simple Dual-Task; SE: Standard Error; YI: Youden Index.
